# Editorial: Screening for Primary Immunodeficiency Disorders (PIDDs) in Neonates

**DOI:** 10.3389/fimmu.2020.633266

**Published:** 2020-12-18

**Authors:** Elham Hossny, Antonio Condino-Neto, Lennart Hammarström, Jolan Eszter Walter

**Affiliations:** ^1^ Pediatric Allergy and Immunology Unit, Children’s Hospital, Ain Shams University, Cairo, Egypt; ^2^ Department of Immunology, Institute of Biomedical Sciences, University of São Paulo, São Paulo, Brazil; ^3^ Division of Clinical Immunology, Department of Laboratory Medicine, Karolinska Institutet at Karolinska University Hospital, Stockholm, Sweden; ^4^ Division of Pediatric Allergy and Immunology, University of South Florida, Tampa, FL, United States; ^5^ Division of Allergy and Immunology, Johns Hopkins All Children’s Hospital, St Petersburg, FL, United States; ^6^ Division of Allergy and Immunology, Massachusetts General Hospital for Children, Boston, MA, United States

**Keywords:** primary immunodeficiency, screening, newborn, neonates, severe combined immunodeficiency

The institution of population-based newborn screening (NBS) has opened a new and exciting era for early diagnosis of primary immunodeficiency disorder (PIDD), which is essential for optimal management of infectious and non-infectious complications leading to high morbidity and mortality. This preventive approach is of high importance for neonates, especially when survival depends on optimal settings for early treatment options such as hematopoietic stem cell transplantation (HSCT). There is an urgent need and opportunity for implementation of neonatal screening for PIDDs globally, as diagnostic tools are developed for SCID and beyond. This Research Topic was aimed to address the current status, limitations and unmet needs of NBS for PIDDs and to draw the attention of immunologists, health care workers and policy makers toward this diagnostic entity in terms of cost effectiveness and global applicability across ethnicities and geographies.

This is a Research Topic collection of 14 articles from several countries around the world. The United States (US) has fully implemented NBS for SCID and now face challenges based on the nationwide variability of the clinical and laboratory approach. Sheller et al. describes the landscape of SCID newborn screening as of 2020, considering the current screening methodologies and targets, communication pathways, and long-term follow-up practices. The authors explore the variation that exists across practices, and emphasize the needs for efficiencies and educational resources in the NBS system to ensure the best outcome. Several papers reported the status of NBS in other geographic locations including data on SCID and other T cell lymphopenia conditions identified among over 130,000 babies screened by T cell receptor excision circles (TRECs) in Catalonia, Spain published by Argudo-Ramírez et al.; and the findings of a group from Hong Kong with Kwok et al., where simultaneous Circles TREC and kappa-deleting recombination excision circles (KREC) quantification were used for detection of a wide variety of PIDD and reference ranges for both KRECs and TRECs were established for distinct age groups. A mini review by El-Sayed and Radwan from Egypt highlights the challenges and major deficits in NBS programs in the developing countries which impedes preventive and curative efforts of managing PIDDs.

NBS for PIDDs other than SCID is gaining momentum with methods based on multiplex protein profiling from dried blood spot samples for parallel diagnosis of 22 innate immunodeficiencies affecting the complement system and respiratory burst function in phagocytes as published by a multinational group (Dezfouli et al.). The proposed method was validated through retrospective screening of immunodeficient patient samples and is applicable for large population-scale performance. Mandola et al. presented the results of a 5-year cohort for diagnosis of Ataxia Telangiectasia (AT) through NBS in Ontario, Canada. They observed a surprisingly high rate of AT through NBS (one vs. five per year for SCID), with distinct genetic variants and ancestry of the patients. The AT patients detected by NBS displayed more profound immunological and neurological phenotype compared to other AT patients. On the same line, Blom et al. from Netherlands reported on a dilemma about diagnosing AT as incidental finding during NBS for SCID from the parents’ perspective. The authors stated that although the current national policy is not to report untreatable incidental findings, unless the health advantage is clear, the majority of parents of healthy neonates in this series were in favor of an early AT diagnosis in the pre-symptomatic phase of the disorder.

Innovations in the methodology of NBS were explored in several articles. Second-tier next generation sequencing (NGS) integrated in the Norwegian nationwide newborn screening program was reported by Strand et al. as means of rapid molecular diagnosis of SCID. Such maneuver on the DNA isolated from the same dried blood spot provided instant confirmation or exclusion of SCID and allowed for the detection of variants of leaky SCID. As a complementary method for SCID-NBS, the multinational European “EuroFlow” standardized approach was proposed by Kalina et al. as unified diagnostic immunopheneotyping for severe PID in children between birth to 2 years of age. The study evaluated the performance of the “SCID-RTE tube” that explores the presence of recent thymic emigrants (RTE) together with T-cell activation status and maturation stages. It was concluded that “EuroFlow SCID-RTE tube” with a previously published PIDOT tube are sensitive and complete cytometric diagnostic test for severe PID (SCID or CID) and for infants identified via NBS with low or absent TRECs. Another multinational study published by Verstegen et al. sought to quantify the T-cell and B-cell replication history in aging, immunodeficiency, and newborn screening. Their results uncovered <5 cell divisions in naive and >10 cell divisions in effector memory T-cell subsets. It also revealed that TREC dilution with age results mainly from increased T cell replication history. Similarly, B cell replication history was higher in patients with primary antibody deficiencies with and without autoimmunity based on KREC assay. The authors propose these assays as second tier to distinguish SCID patients from other PIDs that have false positive NBS for SCID.

Information and emotional support need of families of infants diagnosed as SCID through NBS in the US were explored by Raspa et al. Survey results from parents indicated that the highest-rated information needs were the available treatment options and what to expect across the SCID lifespan. Emotional support needs included dealing with uncertainty about the child’s future and additional opportunities to connect with other families.

Three interesting case reports are included in this Research Topic collection. One of them, by Chitty-Lopez et al., reports a novel hypomorphic variant of the recombination-activating gene (RAG) which was identified by newborn screening in an asymptomatic infant with T cell lymphopenia but preserved B cell count and lymphocyte proliferation. This case highlighted how patients with partial RAG deficiency may present with atypical features as identified by NBS for SCID. Confirmatory functional assays and B cell receptor repertoire studies expedited the process that lead to successful HSCT at 5 months of age. In the other case report, Ricci et al. presented the first case of neuroblastoma amplified sequence deficiency (NBAS) disease detected by NBS for SCID via KREC assay. The authors noted that immune dysfunction, which usually takes the form of severe hypogammaglobulinemia, should never go unnoticed in those infants. Lastly, a novel splice site mutation in the interferon gamma receptor-2 (IFNGR2) gene was reported from India by Bandari et al. in patients exhibiting susceptibility to mycobacterial diseases.

The contributors to this special topic highlighted the targets of neonatal screening being not only severe combined immunodeficiency (SCID) but also some other PIDDs. This shows that research in this topic is escalating and lends further evidence for the cost-effectiveness of this life saving approach. It can pave the way for specific strategies to prevent morbidity and mortality in infants from exposure to early life infections including live vaccines. Newborn screening tests are not regular laboratory tests and their establishment in a country mandates caring for other issues including further investigations in suspected infants to confirm the diagnosis and starting pre-transplant care in the form of providing any required medications, encouraging breast feeding when possible, finding a matched donor, avoiding live vaccines and managing complications of BCG vaccination which is compulsory at birth in some countries. It is also mandatory to organize transplant centers’ network among countries and provide post-transplant follow up and family counseling facilities. The editors hope that this collection of articles would answer some clinical and investigational queries and might stimulate further research in this domain.

## Author Contributions

All authors contributed to the article and approved the submitted version.

## Conflict of Interest

The authors declare that the research was conducted in the absence of any commercial or financial relationships that could be construed as a potential conflict of interest.

